# Knockdown of transient receptor potential melastatin 2 reduces renal fibrosis and inflammation by blocking transforming growth factor-β1-activated JNK1 activation in diabetic mice

**DOI:** 10.18632/aging.203694

**Published:** 2021-11-29

**Authors:** Feng Hu, Yun Yu, Feng Lu, Xiaoshu Cheng

**Affiliations:** 1 The Department of Cardiovascular Medicine, The Second Affiliated Hospital of Nanchang University, Nanchang, China; 2 The Department of Cardiothoracic Surgery, The Second Affiliated Hospital of Nanchang University, Nanchang, China

**Keywords:** diabetic nephropathy, type 2 diabetes, transient receptor potential melastatin 2, renal fibrosis, inflammation

## Abstract

Background: Diabetic nephropathy is a major complication of diabetes. We explore the protective effect of TRPM2 knockdown on the progression of diabetic nephropathy.

Methods: A type 2 diabetes animal model was established in C57BL/6N mice by long-term high-fat diet (HFD) feeding combined with a single injection of 100 mg/kg streptozotocin (STZ). Genetic knockdown of TRPM2 in mouse kidneys was accomplished by the intravenous injection via the tail vein of adeno-associated virus type 2 carrying TRPM2 shRNA.

Results: Mice with HFD/STZ-induced diabetes exhibited kidney dysfunction, as demonstrated by increased blood creatinine and urea nitrogen levels, accompanied by glomerulus derangement, tubule damage and extracellular matrix deposition in the interstitium. The protein expression of TRPM2, transforming growth factor-β1 (TGF-β1), connective tissue growth factor, α-smooth muscles actin, fibronectin, collagen I and collagen III, and the mRNA expression and contents of inflammatory factors, including interleukin-1β, interleukin-6, interferon-α, tumour necrosis factor -α and monocyte chemotactic protein -1, were significantly elevated in the renal tissues of the HFD/STZ-induced diabetes group compared to those of the two control groups. Furthermore, fluorescent staining of TRPM2 was markedly increased in the renal tubular epithelial cells from diabetic mice. Knockdown of TRPM2 significantly attenuated HFD/STZ-induced renal inflammatory responses and fibrosis, which was accompanied by activation of TGF-β1-activated c-Jun N-terminal protein kinase-1 (JNK1) signalling. JNK1 inactivation reversed hyperglycaemia-induced fibrosis and inflammation in HK-2 cells.

Conclusion: TRPM2 silencing significantly attenuated fibrosis and inflammation in the kidneys of mice with HFD/STZ-induced diabetes, which was largely achieved via the inhibition of TGF-β1-activated JNK1 activation.

## INTRODUCTION

The prevalence of diabetes is rapidly increasing owing to the extension of the average life span and the increased prevalence of obesity [1]. Diabetic nephropathy (DN) is a major complication of diabetes, with approximately 40% of diabetic patients presenting with DN [2]. The pathogenesis of DN is currently recognized to be multifactorial, and includes advanced glycation end products [3, 4], hemodynamic changes [5], inflammatory response [6], and autophagy [7], cellular senescence [8], epigenetic regulation [9], and mitochondrial hormesis [10]. Collectively, these changes result in glomerular hyperfiltration and pressure, renal hypertrophy and internal structure derangement [11]. Pathologically, the kidneys undergo several changes, including deposition of extracellular matrix (ECM) proteins, glomerular basement membrane thickening, proliferative changes, and tubular atrophy, ultimately resulting in interstitial fibrosis and glomerulosclerosis [11]. Considering that DN treatment is intractable and complicated, it is urgent to investigate the underlying mechanisms in order to prevent and treat this devastating disease [12].

The transient receptor potential melastatin (TRPM) subfamily contains eight isoforms that form four subfamilies of TRPM channels, namely, TRPM1/3, TRPM2/8, TRPM4/5, and TRPM6/7; these channels play crucial roles in various physiological and pathological conditions, such as conditions related to sensory or renal physiology, cancer, cardiac health and neuronal development [13–16]. Among the isoforms, TRPM7, TRPM2 and TRPM6 are unique because they are bifunctional heteromeric ion channels that contain functional enzymatic domains in their highly diverse C-terminal regions [13–16]. TRPM2 forms a tetrameric cation channel that is permeable to calcium, sodium, and potassium and is activated by oxidative stress through the production of free intracellular adenosine diphosphate-ribose [14, 15]. Its two-in-one protein structure and unique biophysical characteristics enable TRPM2 to be involved in a large number of pathophysiological processes, including cell apoptosis, inflammation and fibrogenesis [14, 15]. For example, NF-κB pathway activation in response to H_2_O_2_ stimulation was blocked in TRPM2-knockout monocytes [17]. Similarly, TRPM2 activation in alveolar epithelial cells was involved in bleomycin-induced pulmonary inflammation [18]. Additionally, inhibition of TRPM2 protects kidneys from ischemia-reperfusion injury by attenuating oxidative stress, inflammation and apoptosis [19]. Furthermore, TRPM2 knockout inhibited fibrosis and inflammation in the kidneys of mice that underwent unilateral urethral obstruction [20]. However, the effect of TRPM2 on the development of DN is unclear.

Here, we discussed whether TRPM2 knockdown has a potential protective effect in the progression of renal inflammation and fibrosis in mice with HFD/STZ-induced diabetes. This study may provide a novel target for preventing and treating DN.

## MATERIALS AND METHODS

### Animal studies

6-week-old male C57BL/6N mice obtained from Hunan SJA Laboratory Animal Co., Ltd (Hunan, China) were fed conventionally at room temperature and optimum humidity under a 12-h light-dark cycle with free access to water and food. All animal experiments were implemented in compliance with the National Institutes of Health (NIH) policies in the Guide for the Care and Use of Laboratory Animals and were approved by the Animal Ethics Committee of the Second Affiliated Hospital of Nanchang University.

We used a high fat diet (HFD) and streptozotocin (STZ, Sigma, USA) injection model to establish T2D as previously described [21–29]. 8-week-old male mice were fed either a HFD (60% of calories from fat, Research Diets, #MD12033, Meidisen, China) or normal chow. After 4 weeks, the HFD-fed mice that exhibited insulin resistance received a single intraperitoneal (i.p.) injection of 100 mg/kg STZ dissolved in 100 mM citrate buffer (pH 4.5) to partly destroy islet function and increase glucose levels. The mice fed normal chow received citrate buffer alone and were processed in parallel with the diabetic mice. All the mice were maintained on their respective diets until the end of the study. The fasting blood glucose (FBG) levels were measured with a blood glucose monitor (ACCU-CHEK Performa, Roche, USA), and the weights of the mice were monitored every per two weeks. Mice with FBG levels > 11.1 mmol/L were considered diabetic and were used for further study [30].

Twenty weeks after STZ treatment [31], all the animals were anaesthetized by an i.p. injection of pentobarbital (55 mg/kg, Sigma) and euthanized by exsanguination. Then, partial renal tissues were fixed in 4% paraformaldehyde for pathological analysis. The remaining tissues were freshly flash-frozen in liquid nitrogen for gene and protein expression analysis.

### Human kidney samples

Formalin-fixed paraffin-embedded (FFPE) specimens of renal biopsy material were obtained from the Pathology Department of the Second Affiliated Hospital of Nanchang University. This study was approved by the Clinical Research Ethics Committee of the Second Affiliated Hospital of Nanchang University. Kidney samples were obtained from the remaining part of diagnostic kidney biopsies of male patients with diabetic nephropathy (n = 6) or normal kidneys (n = 6). Control tissues were deemed normal on routine histological examination. RNA was extracted from archives of FFPE specimens for gene expression analysis.

### Cells culture and grouping

Human kidney-2 (HK-2) cells were obtained from the Cell Bank of Shanghai Chinese Academy of Sciences (SCSP-511, Shanghai, China) and cultured in Dulbecco’s modified Eagle’s medium (DMEM) supplemented with 5.5 mM D-glucose (normal glucose, NG), 10% FBS, 100 U/ml penicillin, and 100 mg/ml streptomycin. The cells were grown to 70–80% before being passaged. Cells at passages three to eight were used in the experiments. In the high-glucose (HG) treatment group, the cells were stimulated with 33 mM D-glucose for 24 h [30].

### Recombinant adeno-associated virus (AAV) construction and infection of mice

The recombinant AAV2-U6-negative control (NC) and recombinant AAV2-U6-shTRPM2 constructs were constructed and ordered from GeneChem (Shanghai, China), and the titers were ~3 × 10^12^ viral genomes per ml (vg/ml). An shRNA sequence targeting TRPM2 (5′-tAACCTTAGCTCATGGATTCCCtcaagagGGGAATCCATGAGCTAAGGTTttttttc-3′), which corresponded to coding regions 68–89 relative to the first nucleotide of the start codon of mouse TRPM2 (GenBank no. NM_138301) and a scrambled (5′-tAATTCTCCGAACGTGTCACGTtcaagagACGTGACACGTTCGGAGAATTttttttc-3′) sequence were cloned into the U6-MCS-CAG-mCherry vector (plasmid GV480).

We knocked down the TRPM2 gene with a single caudal vein injection of 3 ×10^11^ genome copies of recombinant AAV2-U6-shTRPM2 at 8 weeks of age in the corresponding mice. To this end, 32 8-week-old mice were randomly assigned into four groups: (i) AAV2-U6-NC recombinant treated control mice (AAV2-NC group, n = 8); (ii) AAV2-U6-NC recombinant treated mice with HFD/STZ-induced diabetes (AAV2-NC diabetes group, n = 8); (iii) AAV2-U6-shTRPM2 recombinant treated mice (AAV2-shTRPM2 group, n = 8); and (iv) AAV2-U6-shTRPM2 recombinant treated mice with HFD/STZ-induced diabetes (AAV2-shTRPM2 diabetes group, n = 8).

### Pathological tissue morphology

Kidney specimens were perfused, and partial specimens were incubated in 10% neutral formalin fixation solution at room temperature for pathological morphology. These paraffin-embedded sections were stained with hematoxylin-eosin (HE), periodic acid-Schiff (PAS) staining, Sirius Red and Masson staining solutions, respectively [31–33]. Then, the samples were observed with a light microscope (Olympus, Japan). To quantify cardiac fibrosis, 10 fields were randomly selected from 3 cardiac sections, image analysis software (Image-Pro 6.0) was used to calculate the collagen volume fraction (CVF = myocardial collagen area/total area of the image) and the average value was determined.

### Immunofluorescent (IF) staining

5 μm frozen sections of kidney specimens were washed three times with PBS. Then slides were blocked with 5% bull serum albumin (BSA) for half an hour and incubated with both TRPM2 antibody and the antibody of specific renal tubular epithelial cell marker, antiaquaporin 1 (AQP1) overnight at 4° C (Supplementary Table 1). After washing with PBS, slides were correspondingly incubated with two kinds of secondary antibody (Cy3 conjugate, SA00009-1, Proteintech, Wuhan, 1:100; FITC conjugate SA00003-1, Proteintech, Wuhan, 1:100, respectively) for 1 h at room temperature in the darkroom. The cell nuclei were stained with 4',6-diamidino-2-phenylindole (DAPI, C0065, Solarbio, Beijing) for 10 min, and all stained sections were viewed by fluorescent microscope (Olympus, Tokyo).

### Enzyme linked immuno sorbent assay (ELISA) and biochemical determination

Mouse interleukin (IL)-1β, IL-6, interferon (IFN)-α, tumor necrosis factor (TNF)-α and monocyte chemotactic protein (MCP)-1 ELISA kits (MEIMIAN, Shanghai, China) were applied to detect the levels of these cytokine in renal homogenates following the manuals. The levels of blood creatinine and urea nitrogen (BUN) reflected the kidney function using commercial kits purchased from Proteintech Company (Wuhan, China).

### Small interfering RNA (siRNA) transfection and cell grouping

TRPM2 knockdown was achieved by siRNA transfection. si-TRPM2 (sense, 5′-AAGTAGGAGAGGATGTTCAGG-3′; and antisense, 5′-ATCCTCATCCAGTATGTACTC-3′) and Si-negative control (NC, sense, 5′-UUCUCCGAACGUGUCACGUTT-3′; and antisense, 5′-ACGUGACACGUUCGGAGAATT-3) were constructed by Gene Pharma (Shanghai, China). HK-2 cells were instantly transfected with 100 nM si-TRPM2 or Si-NC using GP-transfect-Mate (G04009, GenePharma, Shanghai, China). Western blotting analysis was performed to verify the efficacy of siRNA transfection.

HK-2 cells were instantly transfected with si-TRPM2 or si-NC for 48 h, followed by NG or HG treatment for another 24 h. In another experimental group, HK-2 cells were incubated with 10 μM SP600125 (a JNK inhibitor, S1876, Shanghai, China) for 1 h, followed by HG treatment for 24 h.

### Western blotting

HK-2 cells and homogenized renal specimens were lysed pyrolysised by radioimmunoprecipitation (RIPA) buffer (R0010, Solarbio, Beijing, China) supplemented with cocktail proteinase and phosphatase inhibitors. Protein assay was quantified with the Quantification Kit (PA115, Tiangen, Beijing, China). Equivalent proteins were isolated on sodium dodecylsulphate polyacrylamide gel electrophoresis (SDS-PAGE) and wet transferred onto polyvinylidene fluoride (PVDF) membranes (Millipore, USA). The membranes were blocked in 5% BSA and furthermore cultivated with each primary antibody (Supplementary Table 1) at -4° C overnight. Then, bands were tested by corresponding secondary antibody (Boster, Wuhan, China) and imaged through Pro-light horseradish peroxidase (HRP) chemiluminescence detection reagents (PA112-01, Tiangen, Beijing, China).

### Quantitative real-time polymerase chain reaction (qRT-PCR)

Total RNA was extracted from the kidney samples or cells employing Trizol reagent (Invitrogen Life Technologies, Carlsbad, USA). cDNA synthesis was executed through a TaqMan reverse transcription kit (KR118-03, Tiangen, Beijing, China). Then, qRT-PCR was processed through a SuperReal PreMix Plus Kit (FP205, Tiangen, Beijing, China) on a 7500 FastPCR system from Applied Biosystems (Bio-Rad, USA). The sequences of each primer were presented in Supplementary Table 2. The relative mRNA levels were calculated by the 2^–ΔΔCT^ using GAPDH as a house-keeping gene.

### Statistical analysis

All data were presented as means ± standard deviation (SD) using the Graphpad Prism 8.0 software (GraphPad Software Inc., CA, USA). One-way anova was conducted followed by Bonferroni post hoc test for multiple group comparisons. Statistical analysis was performed using SPSS23.0 (SPSS Inc., Chicago, IL, USA) software and P < 0.05 was considered statistically significant.

### Ethics statement

All animal experiments were conducted in compliance with the National Institutes of Health (NIH) policies in the Guide for the Care and Use of Laboratory Animals and were approved by the Nanchang University Animal Care and Use Committee. The clinical research including human kidney samples was approved by the Clinical Research Ethics Committee of the Second Affiliated Hospital of Nanchang University.

## RESULTS

### TRPM2 expression was increased in the kidneys from mice with HFD/STZ-induced diabetes

**Figure 1:**
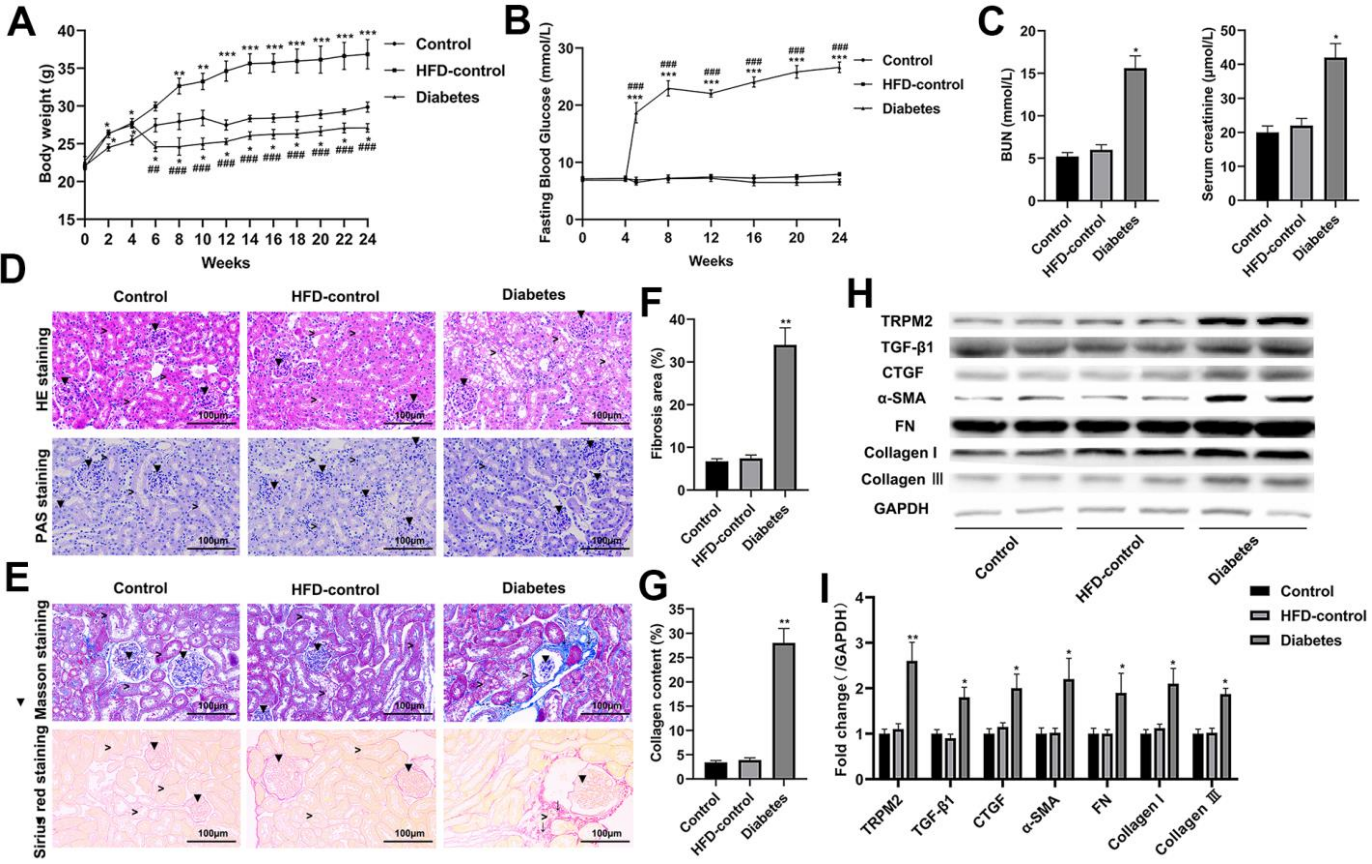
**Renal injury and TRPM2 expression were increased in the kidneys of mice with HFD/STZ-induced diabetes.** (**A**) Body weight of mice was documented from week 0 to 24 of the HFD/STZ-induced diabetes model. (**B**) Blood glucose was measured in the indicated weeks. (**C**) The levels of blood creatinine and BUN were tested before the mice were sacrificed. (**D**) HE and PAS staining of mouse kidney specimens in the 24th week (▼indicates renal glomeruli, ˃ indicated renal tubules). (**E**–**G**) Fibrosis was determined by Masson and Sirius red staining (▼indicates renal glomeruli, ˃ indicates renal tubules, ↓ indicates renal interstitials). (**H**, **I**) Protein analysis was conducted to evaluate the protein expression levels of TRPM2, TGF-β1, CTGF, α-SMA, FN, Collagen I and Collagen III in renal tissues of mice in the 24th week. The data are shown as the mean ± SD, n = 6 per group; *p < 0.05, **p < 0.01 and ***p < 0.001 versus the control group; ^#^p < 0.05, ^##^p < 0.01 and ^###^p < 0.001 versus the HFD-control group.

Compared to the control and HFD-control groups, the mice with HFD/STZ-induced diabetes exhibited hyperglycaemia peaking near 30 mmol/L accompanied by weight loss; these findings indicated metabolic disturbance (Figure 1A, 1B). The mice with HFD/STZ-induced diabetes exhibited kidney dysfunction, as determined by increased serum creatinine and BUN levels (Figure 1C), which were accompanied by glomerulus derangement and proximal tubule damage as shown by HE and PAS staining (Figure 1D). Masson and Sirius staining indicated a severely sclerotic glomerulus, serious tubular damage and collagen accumulation in the vicinity of several large vessels (Figure 1E–1G) in the mice with HFD/STZ-induced diabetes compared to the mice in the other two groups.

**Figure 2:**
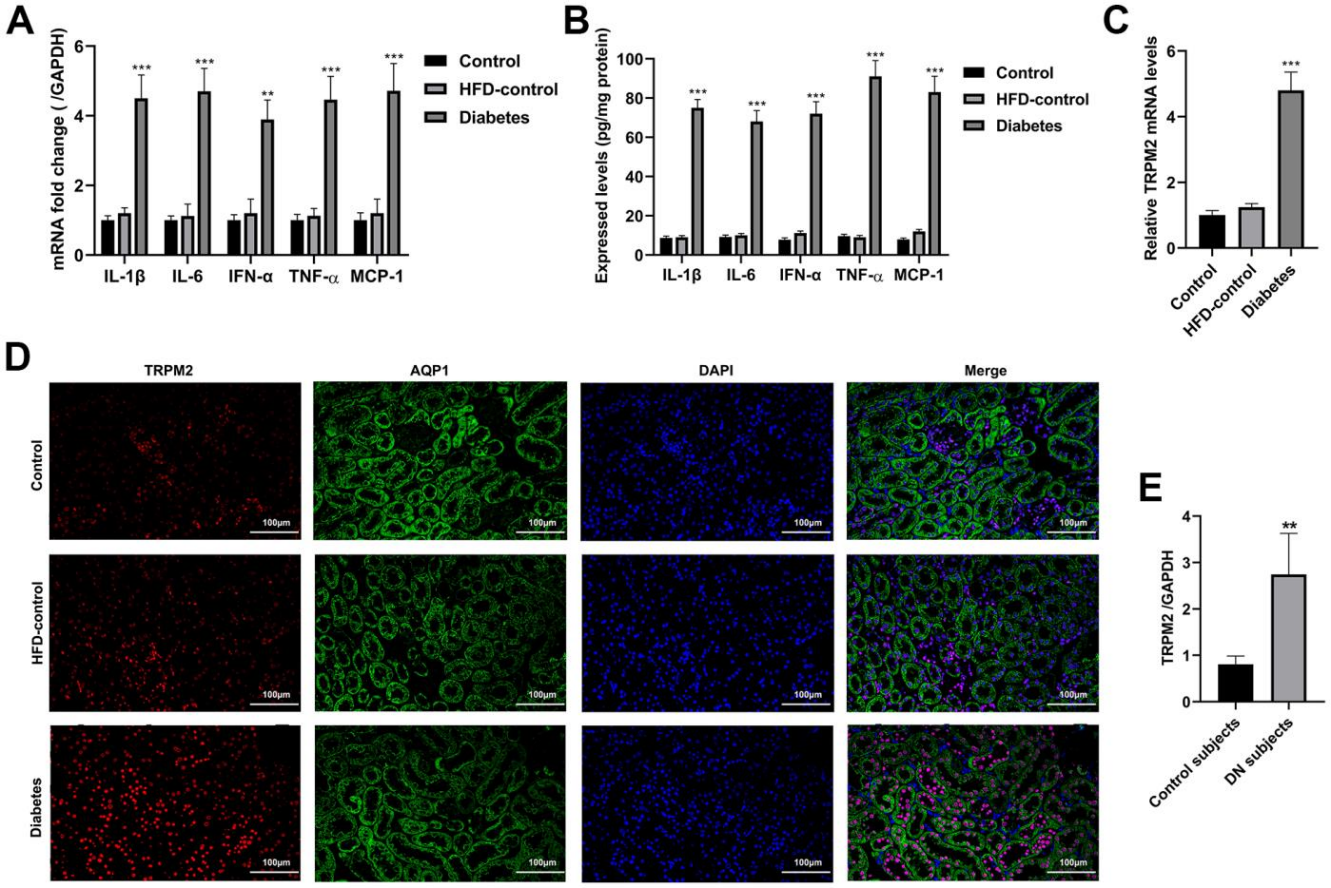
**Inflammatory response and the localization of TRPM2 in the kidneys of mice with HFD/STZ-induced diabetes.** (**A**) qRT-qPCR assay was used to determine the mRNA expression of inflammatory factors including IL-1β, IL-6, IFN-α, TNF-α and MCP-1 in the kidney tissues of mice in the 24th week. (**B**) ELISA was used to determine the contents of inflammatory factors including IL-1β, IL-6, IFN-α, TNF-α and MCP-1 in the kidney tissues of mice in the 24th week. (**C**) qRT-PCR assay was performed to assess the mRNA levels of TRPM2 in the kidney tissues of mice in the 24th week. (**D**) IF double-staining analyses of TRPM2 and APQ1 in the renal tissues of mice in the 24th week. (**E**) qRT-PCR assay was performed to assess the mRNA levels of TRPM2 in kidney biopsies obtained from human subjects with diabetic nephropathy. The data are shown as the mean ± SD, n = 6 per group; *p < 0.05, **p < 0.01 and ***p < 0.001 versus the control group.

Western blotting analysis suggested that the protein expression levels of transforming growth factor-β1 (TGF-β1), connective tissue growth factor (CTGF), α-smooth muscle actin (SMA), fibronectin (FN), collagen I and collagen III were significantly increased in the mice with HFD/STZ-induced diabetes compared to the mice in the other two groups (Figure 1H, 1I). The inflammatory response exacerbates the development of renal injury [34]. The mRNA expression (Figure 2A) and contents (Figure 2B) of cytokines, such as IL-1β, IL-6, IFN-α, TNF-α and MCP-1, were markedly increased in the renal tissues of diabetic mice compared to the renal tissues of the mice in the other two groups.

The mRNA and protein expression levels of TRPM2 were significantly increased in renal specimens from the diabetic mice compared to those from the mice in the other two groups (Figures 1H, 1I, 2C). Hyperglycemia initially damages the glomerulus and successively tubule-interstitial structure in the progress of DN [34]. We next evaluated the localization of TRPM2 in renal tubular epithelial cells. We carried out IF staining utilizing the specific AQP1 marker to identify TRPM2-positive tubular epithelial cells in mouse renal specimens. It is interesting to note that fluorescent staining of TRPM2 was enhanced in the tubular epithelial cells from the kidneys of diabetic mice compared to those from the mice in the other two groups (Figure 2D). Clinically, the mRNA levels of TRPM2 were significantly increased in kidney biopsies obtained from human subjects with diabetic nephropathy (Figure 2E). The characteristics of the human subjects are shown in Supplementary Table 3.

### Knockdown of TRPM2 expression ameliorated renal injury in diabetic mice

**Figure 3:**
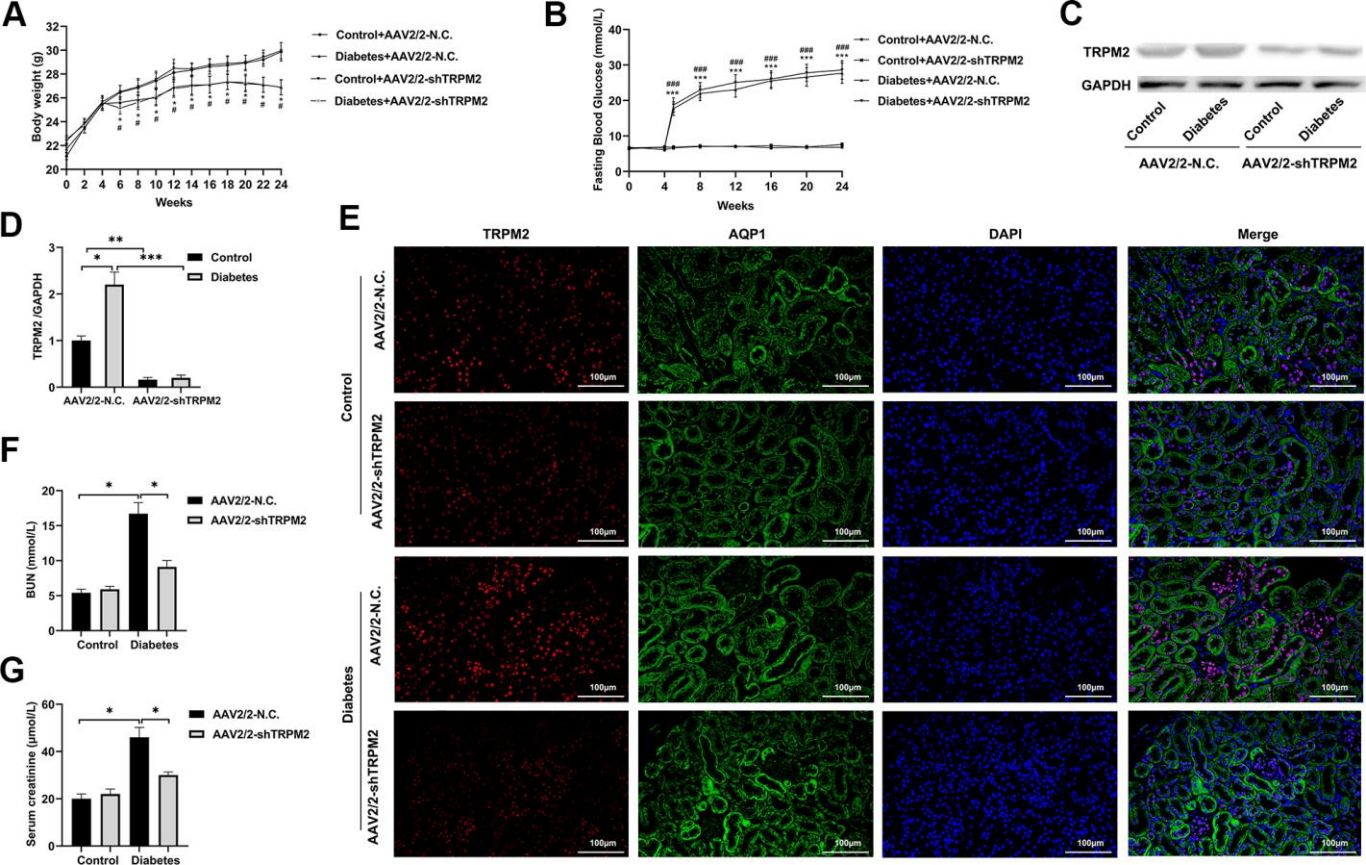
**Silencing TRPM2 attenuated the renal function of diabetic mice.** (**A**) Body weight of mice was documented from week 0 to 24 after the HFD/STZ-induced diabetes model was established. (**B**) Blood glucose levels in the four treatment groups were measured in the indicated weeks. (**C**, **D**) Protein assay of TRPM2 expression in renal specimens from AAV2/2-NC and AAV2/2-shTRPM2 treated mice. (**E**) IF double-staining analyses of TRPM2 and APQ1 in the renal tissues of mice in the 24th week in the four treatment groups. (**F**, **G**) The levels of blood creatinine and BUN were tested before the mice were sacrificed in the four treatment groups. The data are shown as the mean ± SD, n = 6 per group; *p < 0.05, **p < 0.01 and ***p < 0.001. For (**A**, **B**), *p < 0.05 and ***p < 0.001 indicate the AAV2/2-NC + STZ group versus the AAV2/2-NC group, ^#^p < 0.05 and ^###^p < 0.001 indicate the AAV2/2-shTRPM2 + STZ group versus the AAV2/2-shTRPM2 group.

To explore whether TRPM2 knockdown alleviated kidney damage under diabetic conditions, we knocked down TRPM2 expression with by a single caudal vein injection of 3 ×10^11^ genome copies of recombinant AAV2-U6-shTRPM2 at 8 weeks of age in the corresponding mice. AAV2 injection resulted in enhanced expression in the kidneys [35]. We injected AAV2-U6-shTRPM2 recombinant two weeks before starting HFD feeding. Protein quantification confirmed that the level of TRPM2 was effectively reduced in the kidneys of AAV2-shTRPM2 mice (Figure 3C, 3D). In addition, the TRPM2 fluorescence intensity was decreased in the renal tubular epithelial cells from the kidneys of AAV2-shTRPM2 mice (Figure 3E). TRPM2 knockdown by AAV2-shTRPM2 neither changed the levels of FBG (Figure 3B) nor the weight loss in mice with HFD/STZ-induced diabetes (Figure 3A). Nonetheless, TRPM2 knockdown alleviated the HFD/STZ-induced increase in the levels of serum creatinine and BUN, suggesting an improvement in renal function (Figure 3F, 3G).

**Figure 4:**
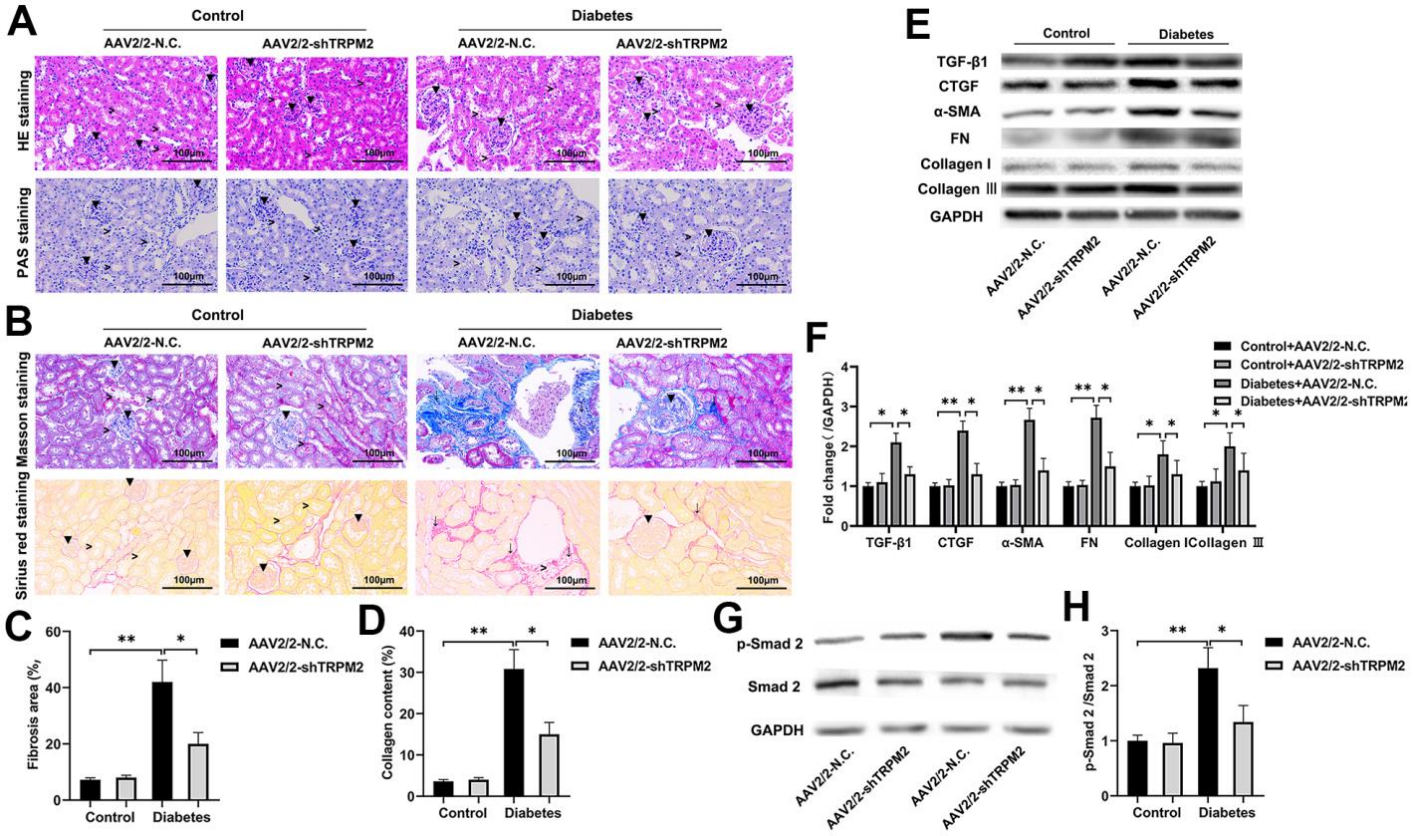
**TRPM2 silencing attenuated renal damage in mice with HFD/STZ-induced diabetes.** (**A**) HE and PAS staining of mouse kidney specimens in the four treatment groups in the 24th week (▼indicates renal glomeruli, ˃ indicates renal tubules). (**B**–**D**) Fibrosis was determined by Masson and Sirius red staining in the four treatment groups (▼ indicates renal glomeruli, ˃ indicates renal tubules, ↓ indicates renal interstitials). (**E**, **F**) Protein assay was implemented to evaluate the protein levels of TGF-β1, CTGF, α-SMA, FN, Collagen I and Collagen III in the renal specimens from the four treatment groups. (**G**, **H**) Protein assays were conducted to evaluate the protein levels of P-Smad2 and Smad2 in the renal specimens of the four treatment groups. The data are shown as the mean ± SD, n = 6 per group; *p < 0.05, **p < 0.01 and ***p < 0.001.

In addition, silencing of TRPM2 attenuated glomerular damage, as shown by HE and PAS staining (Figure 4A), and reduced the severity of glomerular sclerosis, tubular damage and interstitial fibrosis as shown by Masson and Sirius staining (Figure 4B–4D), in the kidneys of diabetic mice. Furthermore, TRPM2 silencing inhibited expression of the pro-fibrotic proteins TGF-β1, p-Smad2, CTGF, α-SMA, FN, Collagen I and Collagen III in the kidneys of diabetic mice (Figure 4E–4H).

### Knockdown of TRPM2 alleviated the renal inflammatory response in diabetic mice

**Figure 5:**
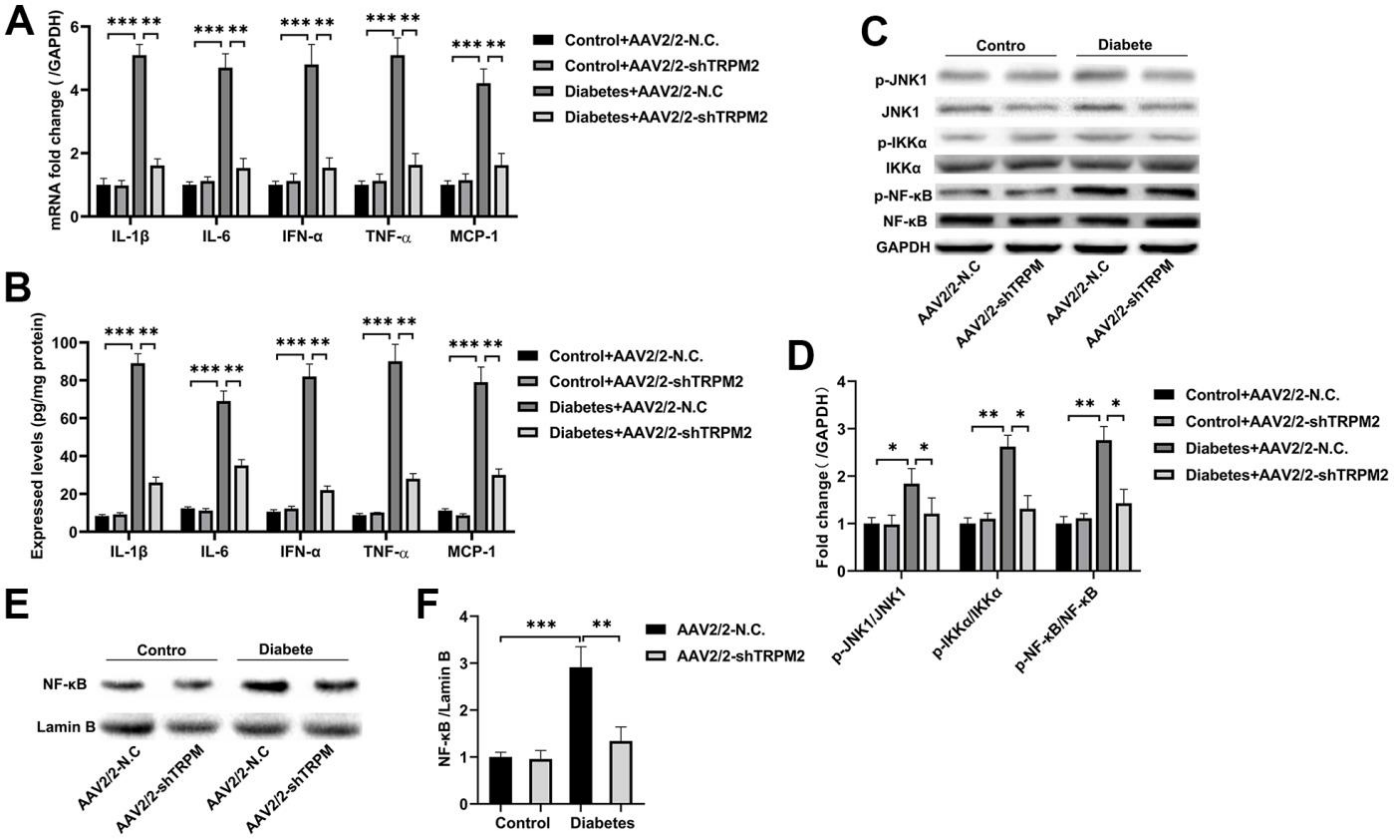
**Silencing TRPM2 alleviated inflammation in the kidneys of mice with HFD/STZ-induced diabetes.** (**A**) qRT-PCR and (**B**) ELISA analysis of the IL-1β, IL-6, IFN-α, TNF-α and MCP-1 levels in the kidney tissues of mice in the four treatment groups in the 24th week. (**C**, **D**) Western blotting assays were conducted to evaluate the protein levels of p-JNK1, p-IKKα and p-NF-κB in the renal specimens of the four treatment groups. (**E**, **F**) Western blotting of the NF-κB levels in the nuclei of the renal specimens in the four treatment groups. The data are shown as the mean ± SD, n = 6 per group; *p < 0.05, **p < 0.01 and ***p < 0.001.

The nosogenesis of DN includes an intractable inflammatory response [36]. The elevated mRNA expression (Figure 5A) and levels (Figure 5B) of inflammatory factors, such as IL-1β, IL-6, IFN-α, TNF-α and MCP-1, in the kidneys of diabetic mice were alleviated by TRPM2 knockdown.

Subsequently, analysis of proteins in the NF-κB pathway showed that TRPM2 knockdown markedly inhibited the levels of p-IKKα and p-NF-κB in the kidneys of diabetic mice (Figure 5C, 5D). The intranuclear aggregation of NF-κB was significantly elevated under the hyperglycemic conditions, whereas its translocation to the nucleus was decreased in TRPM2-silenced mice (Figure 5E, 5F). The above results showed that knockdown of TRPM2 expression might inhibit diabetic inflammatory responses in the kidneys by inhibiting the NF-κB pathway to some degree.

### The role of TRPM2 in c-Jun N-terminal protein kinase (JNK1) activation

JNK signalling plays has an important role in modulating fibrogenesis and inflammation during the development of DN [37]. Therefore, we explored the role of TRPM2 in JNK1 activation during the development of DN. We found that the increased level of p-JNK1 in the kidneys of diabetic mice was inhibited by TRPM2 knockdown (Figure 5C, 5D).

**Figure 6:**
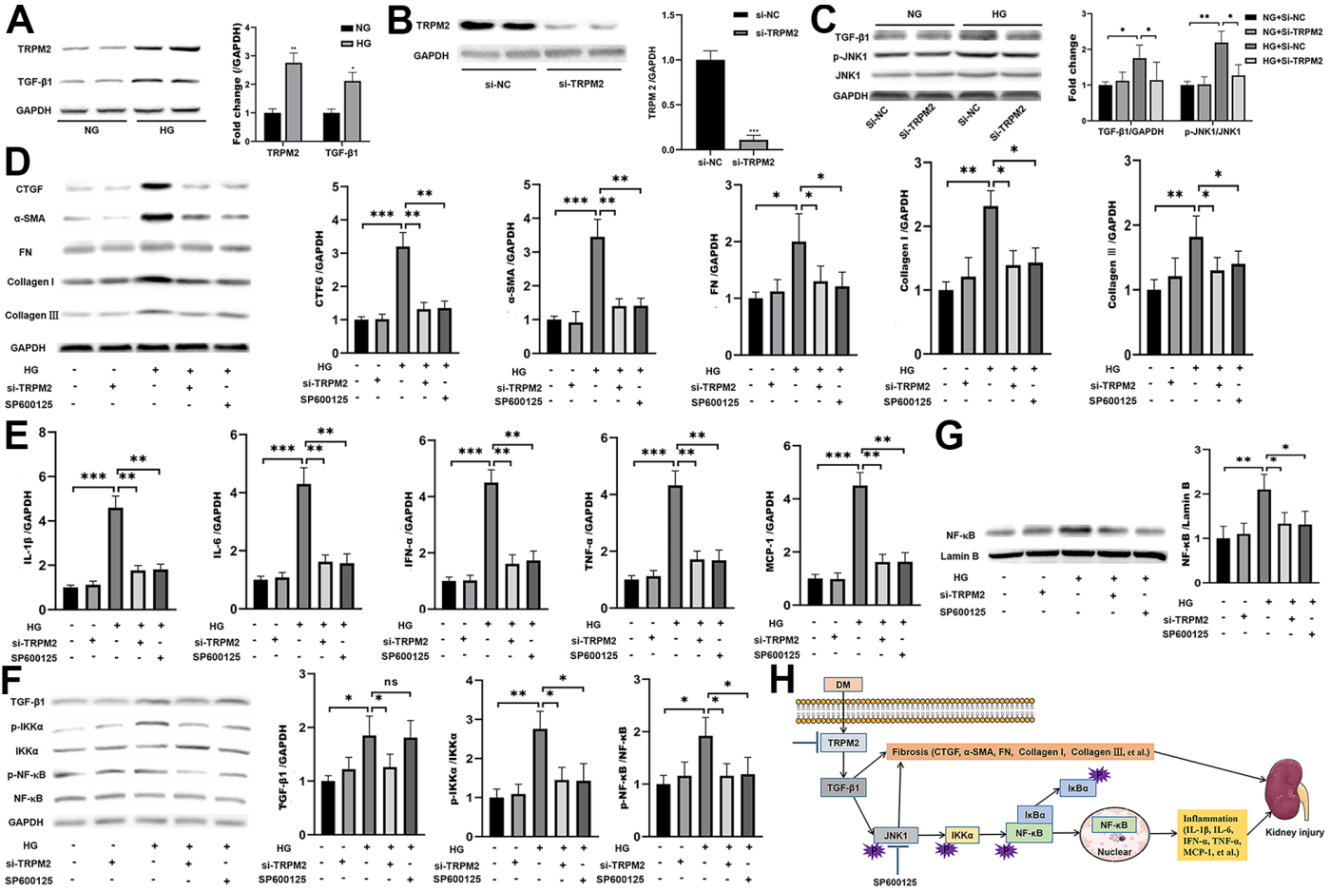
**The protective effect of TRPM2 knockdown in DN by blocking TGF-β1-activated JNK1 activation.** (**A**) Protein assay of TRPM2 and TGF-β1 expression in HK-2 cells stimulated with HG for 24 h. (**B**) Protein analysis was utilized to confirm the efficacy of TRPM2 silencing in HK-2 cells transfected with si-TRPM2. (**C**) HK-2 cells were instantly transfected with si-TRPM2 or Si-NC for 48 h, followed by NG or HG treatment for another 24 h. The protein levels of TGF-β1 and p-JNK1 were detected by protein assay. (**D**) HK-2 cells were instantly transfected with si-TRPM2 or Si-NC for 48 h, followed by NG or HG treatment for another 24 h. In another experimental group, HK-2 cells were incubated with 10 μM SP600125 (a JNK inhibitor) for 1 h, followed by HG treatment for 24 h. Protein assay of CTGF, α-SMA, FN, Collagen I and Collagen III expression in HK-2 cells. (**E**) qRT-PCR analysis of IL-1β, IL-6, IFN-α, TNF-α and MCP-1 expression in HK-2 cells. (**F**) Protein assay of TGF-β1, p-IKKα and p-NF-κB expression in HK2 cells. (**G**) Protein assay of the NF-κB levels in the nuclei of HK2 cells. (**H**) The protective effect of TRPM2 knockdown in DN by blocking TGF-β1-activated JNK1 activation. The data are shown as the mean ± SD of three independent experiments; *p < 0.05, **p < 0.01 and ***p < 0.001.

*In vitro*, HG treatment significantly elevated the protein levels of TRPM2 and TGF-β1 (Figure 6A). Subsequently, TRPM2 expression was downregulated in HK-2 cells using specific si-TRPM2 (Figure 6B). TRPM2 silencing markedly attenuated the protein expression levels of TGF-β1 and p-JNK1 in HG-treated HK-2 cells (Figure 6C). Western blotting analysis showed that HG treatment significantly increased the protein expression levels of CTGF, α-SMA, FN, Collagen I and Collagen III in HK-2 cells, and the expression of these proteins were markedly inhibited by TRPM2 silencing (Figure 6D). In addition, treatment with SP600125 in another experimental group resulted in the downregulation of the HG-induced expression of CTGF, α-SMA, FN, Collagen I and Collagen III (Figure 6D).

As expected, TRPM2 suppression and SP600125 treatment markedly reduced the HG-induced mRNA expression of IL-1β, IL-6, IFN-α, TNF-α and MCP-1 in HK-2 cells (Figure 6E). Moreover, the HG-induced increase in the protein expression levels of cellular p-IKKα p-NF-κB (Figure 6F), and nuclear NF-κB was markedly attenuated by TRPM2 silencing or SP600125 treatment (Figure 6F, 6G). These results showed that TRPM2 activated HG-induced fibrosis and the inflammatory response through the TGF-β1/JNK1 signalling pathway (Figure 6H).

## DISCUSSION

Renal tubule fibrogenesis and inflammation participated in the pathogenesis of chronic renal failure [38]. However, the underlying molecular mechanism has not yet been completely determined. Sustained hyperglycemia with increased fatty acid and cytokine levels gives rise to kidney damage [39]. TRPM2 is a prominent therapeutic target in various chronic maladies [15, 40, 41]. The fundamental purpose of this research was to evaluate whether TRPM2 suppression could be a therapeutic approach for treating DN, and to determine whether renal tubular epithelial cells are involved in the pathological process of DN. Our results showed that TRPM2 expression was elevated under the diabetic/hyperglycemic conditions *in vivo* and *in vitro*. Compared to AAV2/2-NC treatment, silencing TRPM2 alleviated HFD/STZ-induced diabetic renal injury, as shown by ameliorated blood creatinine and BUN levels.

In particular, IF staining showed that the fluorescence intensity of TRPM2 staining was markedly increased in the epithelial tubular cells of kidneys of mice with HFD/STZ-induced diabetes. In addition, HG treatment elevated the levels of fibrotic factors and inflammatory cytokines in HK-2 cells. These results indicated that epithelial cells contributed to the inflammatory response and collagen deposition under the pro-inflammatory and pro-fibrotic conditions associated with DN. Similarly, TRPM2 silencing inhibited the pro-inflammatory and pro-fibrotic processes in HG-treated HK-2 cells. Therefore, these findings suggested that TRPM2 expression in renal tubular epithelial cells participates in the pathogenesis of DN. However, the role of TRPM2 in other renal cell types in the pathogenesis of DN also deserves to be discussed.

TGF-β1 was established to be involved in the progression of DN, and it also exerted fibrogenic effects in this model [42]. Inhibition of the TGF-β1 pathway might be a strategy to prevent the development of renal fibrosis in DN [42]. Hypoxic stress induced TRPM2 expression in adult rat cardiac fibroblasts, resulting in myocardial fibrogenesis [43]. In addition, TRPM2 activated the TGF-β1 signalling pathway to meditate the fibrosis induced by bleomycin [18] and the kidney fibrosis induced by unilateral urethral obstruction [20]. Here, our findings showed that TRPM2 might activate TGF-β1 to aggravate the development of kidney fibrosis in mice with HFD/STZ-induced diabetes.

Numerous studies have shown that the NF-kB participates in the development of inflammatory responses in the kidney triggered by infection, immune response overactivation and hyperglycemia [44, 45]. TRPM2 ubiquitously expressed in different immunocytes, such as neutrophils, monocytes and leukomonocytes [13, 16, 17]. TRPM2 positively increased the nuclear translocation of NF-kB [17]. TRPM2 suppression effectively improved ischemia-reperfusion associated acute kidney injury by inhibiting inflammation [19]. Similarly, TRPM2 silencing ameliorated the inflammatory response of the kidneys of mice that underwent unilateral urethral obstruction by inhibiting TGF-β1-activated JNK activation [20]. TRPM2 knockout improved central nervous system inflammation and cognitive impairment by inhibiting the activation of microglia in a mouse model of chronic cerebral hypoperfusion with bilateral common carotid artery stenosis [46]. Resveratrol ameliorated hypoxia-induced neuronal inflammation by modulating TRPM2 channels [47]. In our research, an inflammatory response was observed in mice with DN as previously described [29, 36]. However, this renal inflammation was reversed by TRPM2 knockdown. Furthermore, in kidney samples from diabetic mice, the expression of p-IKKα and p-NF-κB and the nuclear accumulation of NF-κB were reduced after TRPM2 knockdown. These results indicated that TRPM2 knockdown could relieve inflammatory responses in DN partially by inhibiting the NF-κB pathway.

Extracellular signal regulated kinase (ERK), JNK, and p38-MAPK (mitogen activated protein kinase) are the three subgroups of MAPKs that play significant roles in inflammation, and fibrosis of the kidney [48]. In turn JNK signalling mediates the TGF-β1-induced switch of renal tubular epithelial cells to myofibroblasts [49]. In addition, JNK phosphorylation is closely associated with the exacerbation of inflammation by enhancing the NF-κB pathway [50]. Furthermore, MAPK/NF-κB signaling pathways play a crucial role in the pathogenesis of of DN [29]. TRPM2-mediated chemokine production was associated with JNK pathway activation in lipopolysaccharide-treated microglia [51] and monocytes [17]. TRPM2 knockout markedly lowered inflammation and the fibrotic response by suppressing JNK activation in mice that underwent unilateral urethral obstruction [20]. In our research, JNK1 activation was elevated in DN, which was consistent with previous research results [37, 52]. Hyperglycemia-induced inflammation and fibrosis could be inhibited by TRPM2 silencing through the inhibition of JNK1 activation *in vivo* and *in vitro*. Despite, we could not rule out the possibility that TRPM2-activated renal inflammation and fibrosis might be caused by the activation of p38 and ERK1/2 in mice with HFD/STZ-induced diabetes. Further studies are needed to address this possibility.

This study has some limitations. Considering that knockdown of TRPM2 is not tissue-specific in our study, the significance for finding new therapeutic target is debatable. We will establish renal-specific TRPM2 knockout mice to discuss the protective effect of TRPM2 knockdown on the progression of DN in future. The TRPM2 staining seems to be primarily nuclear, we will explore further the significance of this specific localization.

In conclusion, we showed that TRPM2 silencing significantly ameliorated fibrosis and inflammation in the kidneys of mice with HFD/STZ-induced diabetes, and this effect was largely achieved by the inhibition of TGF-β1-activated JNK1 activation.

## Supplementary Material

Supplementary Tables

## References

[1] Mathers CD, Loncar D. Projections of global mortality and burden of disease from 2002 to 2030. PLoS Med. 2006; 3:e442. 10.1371/journal.pmed.0030442 17132052 PMC1664601

[2] Hu C, Sun L, Xiao L, Han Y, Fu X, Xiong X, Xu X, Liu Y, Yang S, Liu F, Kanwar YS. Insights into the Mechanisms Involved in the Expression and Regulation of Extracellular Matrix Proteins in Diabetic Nephropathy. Curr Med Chem. 2015; 22:2858–70. 10.2174/0929867322666150625095407 26119175 PMC4863711

[3] Papadopoulou-Marketou N, Chrousos GP, Kanaka-Gantenbein C. Diabetic nephropathy in type 1 diabetes: a review of early natural history, pathogenesis, and diagnosis. Diabetes Metab Res Rev. 2017; 33:e2841. 10.1002/dmrr.2841 27457509

[4] Sun HJ, Wu ZY, Cao L, Zhu MY, Liu TT, Guo L, Lin Y, Nie XW, Bian JS. Hydrogen Sulfide: Recent Progression and Perspectives for the Treatment of Diabetic Nephropathy. Molecules. 2019; 24:2857. 10.3390/molecules24152857 31390847 PMC6696501

[5] Lin YC, Chang YH, Yang SY, Wu KD, Chu TS. Update of pathophysiology and management of diabetic kidney disease. J Formos Med Assoc. 2018; 117:662–75. 10.1016/j.jfma.2018.02.007 29486908

[6] Wada J, Makino H. Inflammation and the pathogenesis of diabetic nephropathy. Clin Sci (Lond). 2013; 124:139–52. 10.1042/CS20120198 23075333

[7] Cui J, Bai X, Chen X. Autophagy and Diabetic Nephropathy. Adv Exp Med Biol. 2020; 1207:487–94. 10.1007/978-981-15-4272-5_36 32671771

[8] Xiong Y, Zhou L. The Signaling of Cellular Senescence in Diabetic Nephropathy. Oxid Med Cell Longev. 2019; 2019:7495629. 10.1155/2019/7495629 31687085 PMC6794967

[9] Lu Z, Liu N, Wang F. Epigenetic Regulations in Diabetic Nephropathy. J Diabetes Res. 2017; 2017:7805058. 10.1155/2017/7805058 28401169 PMC5376412

[10] Sharma K. Mitochondrial hormesis and diabetic complications. Diabetes. 2015; 64:663–72. 10.2337/db14-0874 25713188 PMC4338592

[11] Umanath K, Lewis JB. Update on Diabetic Nephropathy: Core Curriculum 2018. Am J Kidney Dis. 2018; 71:884–95. 10.1053/j.ajkd.2017.10.026 29398179

[12] Meza Letelier CE, San Martín Ojeda CA, Ruiz Provoste JJ, Frugone Zaror CJ. [Pathophysiology of diabetic nephropathy: a literature review]. Medwave. 2017; 17:e6839. 10.5867/medwave.2017.01.6839 28112712

[13] Lange I, Penner R, Fleig A, Beck A. Synergistic regulation of endogenous TRPM2 channels by adenine dinucleotides in primary human neutrophils. Cell Calcium. 2008; 44:604–15. 10.1016/j.ceca.2008.05.001 18572241 PMC2597220

[14] Samanta A, Hughes TE, Moiseenkova-Bell VY. Transient Receptor Potential (TRP) Channels. Subcell Biochem. 2018; 87:141–65. 10.1007/978-981-10-7757-9_6 29464560 PMC6038138

[15] Faouzi M, Penner R. TRPM2. Handb Exp Pharmacol. 2014; 222:403–26. 10.1007/978-3-642-54215-2_16 24756715

[16] Carter RN, Tolhurst G, Walmsley G, Vizuete-Forster M, Miller N, Mahaut-Smith MP. Molecular and electrophysiological characterization of transient receptor potential ion channels in the primary murine megakaryocyte. J Physiol. 2006; 576:151–62. 10.1113/jphysiol.2006.113886 16857711 PMC1995624

[17] Yamamoto S, Shimizu S, Kiyonaka S, Takahashi N, Wajima T, Hara Y, Negoro T, Hiroi T, Kiuchi Y, Okada T, Kaneko S, Lange I, Fleig A, et al. TRPM2-mediated Ca2+influx induces chemokine production in monocytes that aggravates inflammatory neutrophil infiltration. Nat Med. 2008; 14:738–47. 10.1038/nm1758 18542050 PMC2789807

[18] Yonezawa R, Yamamoto S, Takenaka M, Kage Y, Negoro T, Toda T, Ohbayashi M, Numata T, Nakano Y, Yamamoto T, Mori Y, Ishii M, Shimizu S. TRPM2 channels in alveolar epithelial cells mediate bleomycin-induced lung inflammation. Free Radic Biol Med. 2016; 90:101–13. 10.1016/j.freeradbiomed.2015.11.021 26600069

[19] Eraslan E, Tanyeli A, Polat E, Polat E. 8-Br-cADPR, a TRPM2 ion channel antagonist, inhibits renal ischemia-reperfusion injury. J Cell Physiol. 2019; 234:4572–81. 10.1002/jcp.27236 30191993

[20] Wang Y, Chen L, Wang K, Da Y, Zhou M, Yan H, Zheng D, Zhong S, Cai S, Zhu H, Li Y. Suppression of TRPM2 reduces renal fibrosis and inflammation through blocking TGF-β1-regulated JNK activation. Biomed Pharmacother. 2019; 120:109556. 10.1016/j.biopha.2019.109556 31655312

[21] Rendina-Ruedy E, Hembree KD, Sasaki A, Davis MR, Lightfoot SA, Clarke SL, Lucas EA, Smith BJ. A Comparative Study of the Metabolic and Skeletal Response of C57BL/6J and C57BL/6N Mice in a Diet-Induced Model of Type 2 Diabetes. J Nutr Metab. 2015; 2015:758080. 10.1155/2015/758080 26146567 PMC4469802

[22] Li H, Li Y, Xiang L, Zhang J, Zhu B, Xiang L, Dong J, Liu M, Xiang G. GDF11 Attenuates Development of Type 2 Diabetes via Improvement of Islet β-Cell Function and Survival. Diabetes. 2017; 66:1914–27. 10.2337/db17-0086 28450417

[23] Kusakabe T, Tanioka H, Ebihara K, Hirata M, Miyamoto L, Miyanaga F, Hige H, Aotani D, Fujisawa T, Masuzaki H, Hosoda K, Nakao K. Beneficial effects of leptin on glycaemic and lipid control in a mouse model of type 2 diabetes with increased adiposity induced by streptozotocin and a high-fat diet. Diabetologia. 2009; 52:675–83. 10.1007/s00125-009-1258-2 19169663

[24] Lee JY, Jeong EA, Kim KE, Yi CO, Jin Z, Lee JE, Lee DH, Kim HJ, Kang SS, Cho GJ, Choi WS, Choi SY, Kwon HM, Roh GS. TonEBP/NFAT5 haploinsufficiency attenuates hippocampal inflammation in high-fat diet/streptozotocin-induced diabetic mice. Sci Rep. 2017; 7:7837. 10.1038/s41598-017-08319-w 28798347 PMC5552681

[25] Yu L, Liang Q, Zhang W, Liao M, Wen M, Zhan B, Bao H, Cheng X. HSP22 suppresses diabetes-induced endothelial injury by inhibiting mitochondrial reactive oxygen species formation. Redox Biol. 2019; 21:101095. 10.1016/j.redox.2018.101095 30640127 PMC6327915

[26] Mátyás C, Németh BT, Oláh A, Török M, Ruppert M, Kellermayer D, Barta BA, Szabó G, Kökény G, Horváth EM, Bódi B, Papp Z, Merkely B, Radovits T. Prevention of the development of heart failure with preserved ejection fraction by the phosphodiesterase-5A inhibitor vardenafil in rats with type 2 diabetes. Eur J Heart Fail. 2017; 19:326–36. 10.1002/ejhf.711 27995696 PMC5347963

[27] Teng X, Ji C, Zhong H, Zheng D, Ni R, Hill DJ, Xiong S, Fan GC, Greer PA, Shen Z, Peng T. Selective deletion of endothelial cell calpain in mice reduces diabetic cardiomyopathy by improving angiogenesis. Diabetologia. 2019; 62:860–72. 10.1007/s00125-019-4828-y 30778623 PMC6702672

[28] Wang X, Pan J, Liu H, Zhang M, Liu D, Lu L, Tian J, Liu M, Jin T, An F. AIM2 gene silencing attenuates diabetic cardiomyopathy in type 2 diabetic rat model. Life Sci. 2019; 221:249–58. 10.1016/j.lfs.2019.02.035 30790610

[29] Li Y, Hou JG, Liu Z, Gong XJ, Hu JN, Wang YP, Liu WC, Lin XH, Wang Z, Li W. Alleviative effects of 20(R)-Rg3 on HFD/STZ-induced diabetic nephropathy via MAPK/NF-κB signaling pathways in C57BL/6 mice. J Ethnopharmacol. 2021; 267:113500. 10.1016/j.jep.2020.113500 33091499

[30] Zheng C, Huang L, Luo W, Yu W, Hu X, Guan X, Cai Y, Zou C, Yin H, Xu Z, Liang G, Wang Y. Inhibition of STAT3 in tubular epithelial cells prevents kidney fibrosis and nephropathy in STZ-induced diabetic mice. Cell Death Dis. 2019; 10:848. 10.1038/s41419-019-2085-0 31699972 PMC6838321

[31] Rodionov RN, Jarzebska N, Schneider A, Rexin A, Sradnick J, Brilloff S, Martens-Lobenhoffer J, Bode-Böger SM, Todorov V, Hugo C, Weiss N, Hohenstein B. ADMA elevation does not exacerbate development of diabetic nephropathy in mice with streptozotocin-induced diabetes mellitus. Atheroscler Suppl. 2019; 40:100–05. 10.1016/j.atherosclerosissup.2019.08.040 31818438

[32] Liang G, Song L, Chen Z, Qian Y, Xie J, Zhao L, Lin Q, Zhu G, Tan Y, Li X, Mohammadi M, Huang Z. Fibroblast growth factor 1 ameliorates diabetic nephropathy by an anti-inflammatory mechanism. Kidney Int. 2018; 93:95–109. 10.1016/j.kint.2017.05.013 28750927 PMC5818994

[33] Gong W, Chen Z, Zou Y, Zhang L, Huang J, Liu P, Huang H. CKIP-1 affects the polyubiquitination of Nrf2 and Keap1 via mediating Smurf1 to resist HG-induced renal fibrosis in GMCs and diabetic mice kidneys. Free Radic Biol Med. 2018; 115:338–50. 10.1016/j.freeradbiomed.2017.12.013 29248720

[34] Silverstein DM. Inflammation in chronic kidney disease: role in the progression of renal and cardiovascular disease. Pediatr Nephrol. 2009; 24:1445–52. 10.1007/s00467-008-1046-0 19083024

[35] Qi YF, Li QH, Shenoy V, Zingler M, Jun JY, Verma A, Katovich MJ, Raizada MK. Comparison of the transduction efficiency of tyrosine-mutant adeno-associated virus serotype vectors in kidney. Clin Exp Pharmacol Physiol. 2013; 40:53–55. 10.1111/1440-1681.12037 23216315 PMC3621769

[36] Ni Z, Guo L, Liu F, Olatunji OJ, Yin M. Allium tuberosum alleviates diabetic nephropathy by supressing hyperglycemia-induced oxidative stress and inflammation in high fat diet/streptozotocin treated rats. Biomed Pharmacother. 2019; 112:108678. 10.1016/j.biopha.2019.108678 30784905

[37] Yang H, Kan QE, Su Y, Man H. Long Non-Coding RNA CASC2 Improves Diabetic Nephropathy by Inhibiting JNK Pathway. Exp Clin Endocrinol Diabetes. 2019; 127:533–37. 10.1055/a-0629-9958 29890555

[38] Sato M, Muragaki Y, Saika S, Roberts AB, Ooshima A. Targeted disruption of TGF-beta1/Smad3 signaling protects against renal tubulointerstitial fibrosis induced by unilateral ureteral obstruction. J Clin Invest. 2003; 112:1486–94. 10.1172/JCI19270 14617750 PMC259132

[39] Mathur A, Pandey VK, Kakkar P. Activation of GSK3β/β-TrCP axis via PHLPP1 exacerbates Nrf2 degradation leading to impairment in cell survival pathway during diabetic nephropathy. Free Radic Biol Med. 2018; 120:414–24. 10.1016/j.freeradbiomed.2018.04.550 29655866

[40] Miller BA. TRPM2 in Cancer. Cell Calcium. 2019; 80:8–17. 10.1016/j.ceca.2019.03.002 30925291 PMC6545160

[41] Belrose JC, Jackson MF. TRPM2: a candidate therapeutic target for treating neurological diseases. Acta Pharmacol Sin. 2018; 39:722–32. 10.1038/aps.2018.31 29671419 PMC5943913

[42] Chang AS, Hathaway CK, Smithies O, Kakoki M. Transforming growth factor-β1 and diabetic nephropathy. Am J Physiol Renal Physiol. 2016; 310:F689–96. 10.1152/ajprenal.00502.2015 26719364 PMC4835922

[43] Takahashi K, Sakamoto K, Kimura J. Hypoxic stress induces transient receptor potential melastatin 2 (TRPM2) channel expression in adult rat cardiac fibroblasts. J Pharmacol Sci. 2012; 118:186–97. 10.1254/jphs.11128fp 22293297

[44] Zhang H, Sun SC. NF-κB in inflammation and renal diseases. Cell Biosci. 2015; 5:63. 10.1186/s13578-015-0056-4 26579219 PMC4647710

[45] Tamada S, Asai T, Kuwabara N, Iwai T, Uchida J, Teramoto K, Kaneda N, Yukimura T, Komiya T, Nakatani T, Miura K. Molecular mechanisms and therapeutic strategies of chronic renal injury: the role of nuclear factor kappaB activation in the development of renal fibrosis. J Pharmacol Sci. 2006; 100:17–21. 10.1254/jphs.fmj05003x4 16397373

[46] Miyanohara J, Kakae M, Nagayasu K, Nakagawa T, Mori Y, Arai K, Shirakawa H, Kaneko S. TRPM2 Channel Aggravates CNS Inflammation and Cognitive Impairment via Activation of Microglia in Chronic Cerebral Hypoperfusion. J Neurosci. 2018; 38:3520–33. 10.1523/JNEUROSCI.2451-17.2018 29507145 PMC6596050

[47] Akyuva Y, Nazıroğlu M. Resveratrol attenuates hypoxia-induced neuronal cell death, inflammation and mitochondrial oxidative stress by modulation of TRPM2 channel. Sci Rep. 2020; 10:6449. 10.1038/s41598-020-63577-5 32296107 PMC7160154

[48] Kyriakis JM, Avruch J. Mammalian MAPK signal transduction pathways activated by stress and inflammation: a 10-year update. Physiol Rev. 2012; 92:689–737. 10.1152/physrev.00028.2011 22535895

[49] Lan R, Geng H, Polichnowski AJ, Singha PK, Saikumar P, McEwen DG, Griffin KA, Koesters R, Weinberg JM, Bidani AK, Kriz W, Venkatachalam MA. PTEN loss defines a TGF-β-induced tubule phenotype of failed differentiation and JNK signaling during renal fibrosis. Am J Physiol Renal Physiol. 2012; 302:F1210–23. 10.1152/ajprenal.00660.2011 22301622 PMC3362177

[50] Liu SH, Lu TH, Su CC, Lay IS, Lin HY, Fang KM, Ho TJ, Chen KL, Su YC, Chiang WC, Chen YW. Lotus leaf (Nelumbo nucifera) and its active constituents prevent inflammatory responses in macrophages via JNK/NF-κB signaling pathway. Am J Chin Med. 2014; 42:869–89. 10.1142/S0192415X14500554 25004880

[51] Biernacki M, Ambrożewicz E, Gęgotek A, Toczek M, Bielawska K, Skrzydlewska E. Redox system and phospholipid metabolism in the kidney of hypertensive rats after FAAH inhibitor URB597 administration. Redox Biol. 2018; 15:41–50. 10.1016/j.redox.2017.11.022 29197803 PMC5723275

[52] Sheng J, Li H, Dai Q, Lu C, Xu M, Zhang J, Feng J. DUSP1 recuses diabetic nephropathy via repressing JNK-Mff-mitochondrial fission pathways. J Cell Physiol. 2019; 234:3043–57. 10.1002/jcp.27124 30191967

