# Supplementary material for: Knockdown of transient receptor potential melastatin 2 reduces renal fibrosis and inflammation by blocking transforming growth factor-β1-activated JNK1 activation in diabetic mice
Source: Aging (Albany NY). 2021 Nov 29;13(22):24605–20. doi: 10.18632/aging.203694 (PMC8660601; doi:10.18632/aging.203694)
Supplement: Supplementary Tables [file aging-13-203694-s001.pdf]

SUPPLEMENTARY TABLES

Supplementary Table 1. List of primary antibodies.

Name	Catalog	Analysis	Dilutions	Company
TRPM2	#PA1-46473	Western blot<br>IF staining	1:1000<br>1:50	Invitrogen
Antiaquaporin 1(AQP1)	#NB600-749	IF staining	1:200	Novus Biologicals
TGF-β1	#ab64715	Western blot	1:1000	Abcam
CTGF	#bs-0743R	Western blot	1:1000	Bioss
α-SMA	#bs-33187M	Western blot	1:1000	Bioss
Fibronectin	#bs-0666R	Western blot	1:1000	Bioss
Collagen I	#bs-7158R	Western blot	1:500	Bioss
Collagen III	#bs-0549P	Western blot	1:1000	Bioss
Phospho-AMPK(S487)	#ab131357	Western blot	1:1000	Abcam
JNK1	#ab199380	Western blot	1:1000	Abcam
Phospho-IKKα (T23)	#32041	Western blot	1:1000	Abcam
IKKα	#ab32041	Western blot	1:1000	Abcam
Phospho-NF-κB (Ser536)	#3033	Western blot	1:1000	CST
NF-κB	#8242	Western blot	1:1000	CST
Lamin B	#ab32535	Western blot	1:500	Abcam
GAPDH	#60004	Western blot	1:10000	Proteintech

IF, immunofluorescence.

Supplementary Table 2. The RT-qPCR sequences used in the study.

Gene name	Primer sequences (5'→3')
mouse-TRPM2	F: TACTCGCTTACGTGCGTAG<br>R: ATCGCTTCCGCCATAGTT
human-TRPM2	GAAGAGCATTTTCCGCAGAG<br>ATGAGCTCGCCTTCCTTGTA
mouse-TNF-α	F:CATCTTCTCAAAACTCGAGTGACAA<br>R: TGGGAGTAGATAAGGTACAGCCC
mouse-IL-1β	F: GCCTCGTGCTGTCGGACCCATAT<br>R: TCCTTTGAGGCCCAAGGCCACA
mouse-IL-6	F: TCCGAGTTGCCGAACGAC<br>R: ATGACTCTATGCTCCACGTC
mouse-MCP-1	F: CCCACTCACCTGCTGCTACT<br>R: TCTGGACCCATTCCTTCTTG
mouse- IFN-γ	F: GCGTCATTGAATCACACCTG<br>R: TGAGCTCATTGAATGCTTGG
mouse-GAPDH	F: AGGTCGGTGTGAACGGATTTG<br>R: TGTAGACCATGTAGTTGAGGTCA
human-GAPDH	F: CTGCACCACCAACTGCTTAG<br>R: AGGTCCACCACTGACACGTT

Supplementary Table 3. Clinical data on male patient samples.

	Normal	Diabetic nephropathy	P-value
Total numbers	6	6	
Age	54.33 ± 2.93	54.67 ± 2.91	0.937
Weight (kg)	72.20 ± 3.57	62.20 ± 2.58	0.047
Body mass index (kg/m^2)	24.38 ± 0.62	20.85 ± 1.09	0.019
Systolic blood pressure on admission (mmHg)	115.50 ± 4.32	157.17 ± 3.73	<0.001
Diastolic blood pressure on admission (mmHg)	73.17 ± 1.54	84.00 ± 1.93	0.001
Serum creatinine (mmol/L)	63.83 ± 6.45	148.83 ± 20.66	0.008
Serum uric acid (umol/L)	231.00 ± 12.56	370.50 ± 22.62	<0.001
Estimated glomerular filtration rate ($ml/min/1.73m^2$)	108.00 ± 3.30	58.83 ± 2.34	<0.001
Serum albumin (g/L)	38.17 ± 3.50	26.33 ± 1.98	0.015
Proteinuria (mg/day)	59.50 ± 4.82	3620.00 ± 613.89	<0.001

Data are means ± S.E.
